# The role of miRNAs in stress-responsive hepatic stellate cells during liver fibrosis

**DOI:** 10.3389/fphys.2015.00209

**Published:** 2015-07-28

**Authors:** Joeri Lambrecht, Inge Mannaerts, Leo A. van Grunsven

**Affiliations:** Liver Cell Biology Lab, Department of Biomedical Sciences, Vrije Universiteit BrusselBrussels, Belgium

**Keywords:** miRNAs, hepatic stellate cells, fibrosis, ER stress, hypoxia, oxidative stress

## Abstract

The progression of liver fibrosis and cirrhosis is associated with the persistence of an injury causing agent, leading to changes in the extracellular environment and a disruption of the cellular homeostasis of liver resident cells. Recruitment of inflammatory cells, apoptosis of hepatocytes, and changes in liver microvasculature are some examples of changing cellular environment that lead to the induction of stress responses in nearby cells. During liver fibrosis, the major stresses include hypoxia, oxidative stress, and endoplasmic reticulum stress. When hepatic stellate cells (HSCs) are subjected to such stress, they modulate fibrosis progression by induction of their activation toward a myofibroblastic phenotype, or by undergoing apoptosis, and thus helping fibrosis resolution. It is widely accepted that microRNAs are import regulators of gene expression, both during normal cellular homeostasis, as well as in pathologic conditions. MicroRNAs are short RNA sequences that regulate the gene expression by mRNA destabilization and inhibition of mRNA translation. Specific microRNAs have been identified to play a role in the activation process of HSCs on the one hand and in stress-responsive pathways on the other hand in other cell types (**Table 2**). However, so far there are no reports for the involvement of miRNAs in the different stress responses linked to HSC activation. Here, we review briefly the major stress response pathways and propose several miRNAs to be regulated by these stress responsive pathways in activating HSCs, and discuss their potential specific pro-or anti-fibrotic characteristics.

## Introduction

Liver fibrosis is the pathological condition of the liver resulting from sustained wound healing in response to chronic liver injury. Multiple factors can lead to such injury, including genetic (the accumulation of misfolded alpha1-antitrypsin), cholestatic (sclerosing cholangitis), metabolic (non-alcoholic fatty liver disease and non-alcoholic steatohepatitis), drug induced (paracetamol-intoxication and alcohol) and viral diseases (hepatitis B and C) (Friedman, 2003; Wallace et al., 2008). Liver fibrosis can eventually progress toward cirrhosis, which is characterized by the loss of endothelial fenestrations, excessive scar formation in the space of Disse, and the presence of vascularized fibrotic septa. These distortions of liver architecture and subsequent cellular homeostasis lead to impaired organ function, ascites, encephalopathy, variceal hemorrhage, portal hypertension and the development of hepatocellular carcinoma (Schuppan and Afdhal, 2008).

## Role of miRNAs during hepatic stellate cell activation

One of the key features in the development of liver fibrosis is the augmenting presence of myofibroblasts in the liver. Myofibroblasts are characterized by their stellate shape, the expression of some specific proteins, such as alpha-smooth muscle actin (α-SMA), and the excessive production of extracellular matrix proteins, including fibronectin and collagen type I, III, and IV. Hepatic stellate cells (HSCs) transdifferentiate upon injury into myofibroblasts, and can be considered as the major origin of myofibroblasts (Mederacke et al., 2013). During initiation and progression of the liver fibrosis process, the liver is subjected to various kinds of stress including hypoxia (Nath and Szabo, 2012), oxidative stress (Parola and Robino, 2001), and endoplasmic reticulum (ER) stress (Li et al., 2015). HSCs will respond by activating into myofibroblasts, which is characterized by a change in gene (Jiang et al., 2006; De Minicis et al., 2007) and microRNA expression (Guo et al., 2009a), as reviewed in He et al. (2012a); Huang et al. (2014a) and Coll et al. (2015). Numerous detailed reports on gene expression changes during HSC activation are available, but information regarding their regulation by specific miRNAs remains rather vague.

**Figure 1:**
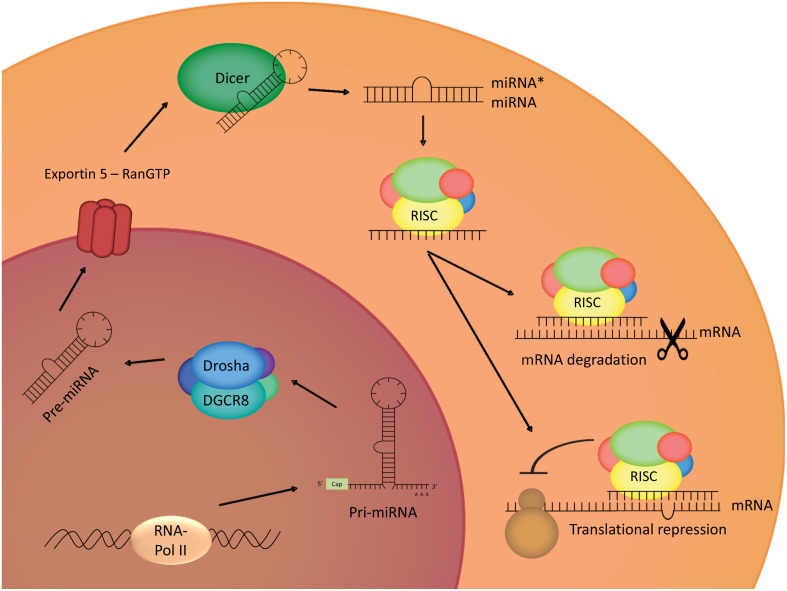
**MiRNA biogenesis**. Transcription of the genes coding for miRNAs leads to the generation of primary miRNAs, which will be cleaved in the nucleus by Drosha, a ribonuclease III complex. The produced ribonucleic structure is called premature miRNA, and will be transported to the cytoplasm by Exportin 5, where it will undergo cleaving by Dicer, another ribonuclease III enzyme. One strain of the double-stranded obtained structure will integrate in the RISC-complex, leading to translational repression, or degradation of the target mRNA.

MiRNAs are short non-protein coding RNA sequences of 20–23 nucleotides that are evolutionary conserved and are encoded in the genome. The human genome is supposed to encode for approximately 1000 miRNAs, which can be expressed in an ubiquitous or a tissue/cell-type specific way (Lee, 2013), and each of these miRNAs is thought to have a great range of potential targets, thus indicating its importance in gene regulation (Bartel and Chen, 2004). MiRNA-encoding genes are transcribed by RNA polymerase II, with the generation of primary miRNA, which will then be processed in the nucleus by activity of a microprocessor complex, named Drosha. The activity of this Drosha containing complex leads to the production of a hairpin-shaped premature miRNA defined by a length of approximately 70 nucleotides and the presence of a stem-loop structure (Lee et al., 2003; Gregory et al., 2004). Correctly processed premature miRNAs are then bound by Exportin-5 in a Ran guanosine triphosphate (RanGTP)-dependent manner, leading to the transport of these pre-miRNAs toward the cytoplasm (Lund et al., 2004). In the cytoplasm, the pre-miRNAs undergo processing by Dicer, another ribonuclease III enzyme, resulting in the production of double stranded RNA (dsRNA) of 20–23 nucleotides (Bernstein et al., 2001). In this double stranded nucleotide-complex, a mature miRNA strand, known as the guide strand, and a miRNA^*^ strand, known as the passenger strand can be identified. The mature miRNA strand will be loaded into the Argonaute 2 (Ago2)-containing RNA-induced silencing complex (RISC), which is the effector of miRNA-mediated activities (Gregory et al., 2005). It is believed that the RISC complex can cause down-regulation of gene expression through 2 mechanisms; by an inhibition of mRNA translation or by reducing the mRNA stability and thus facilitating the degradation (Figure 1) (Bagga et al., 2005; Orban and Izaurralde, 2005; Pillai et al., 2005).

Since the discovery of miRNAs in 1993 (Wightman et al., 1993), researchers continuously tried to evoke the role of miRNAs in cellular homeostasis and in development of pathological conditions, including liver fibrosis. There are many miRNAs expressed during, and described to be involved in, HSC activation (Table 1), making them the topic of concise reviews (He et al., 2012a; Huang et al., 2014a). Here, we only briefly highlight some key miRNAs to illustrate the possible roles a miRNA could have in quiescent or activated HSCs. When evaluating these miRNA-studies it is important to keep in mind that although many miRNAs are conserved among eukaryotic organisms, it is possible that they do not display the same expression patterns in specific (pathological) processes, and thus can display interspecies differences in expression (Ha et al., 2008).

**Table 1:** **Significantly regulated miRNAs during HSC-activation**.

**References**	**Up-regulated**	**Down-regulated**
**MiRNAs REGULATED DURING HSC ACTIVATION**		
Guo et al., 2009b	miR−29c^*^, −138, −140, −**143**, −**193**, −207, −325−5p, −328, −349, −501, −872, −874	miR−15, −**16**, −20*b*−3p, −92b, −122, −**126**, −**146a**, −341, −375
Ji et al., 2009	miR-27a, −27b, −30a, −30c, −30d, −130a, −130b, −450, −455	miR-9, −**19b**, −301, −520b, −520c, −721
Maubach et al., 2011	Let-7b, −7c, −7e, miR-**125b**, −21, −22, −31, −132, −**143**, −**145**, −**152**, −**199a**, −210, −**214**, −**221**, −**222**	Let-7f, miR−**10a**, −16, −26b, −**29a**, −30*a*−5p, −30b, −30c, −30d, −99a, −122a, −125a, −**126**, −**146a**, −**150**, −151^*^, −181a, −**192**, −**194**, −**195**, −207, −296, −**335**, −422b, −483
Chen et al., 2011	miR-31, −34b, −**34c**, −**125b**−**5p**, −**143**, −**145**, −**152**, −**193**, −**199a**−**5p**, −199a−3p, −**214**, −218, −**221**, −**222**, −301a,−345−5p, −425	miR-**10a**−**5p**, −101a, −**126**, −126^*^, −139−5p, −**150**, −**192**, −**195**, −**335**, −338, −378^*^, −450a, −497, −877
Lakner et al., 2012	miR−34**c**, −184, −**221**	miR−**16**, −19a, −**19b**, −29**a**, −29c, −92a, −150, −194

*Summary of published data regarding microRNA microarray profiling of activating primary rat HSCs. MiRNAs which display an overlap in different published data sets are displayed in bold*.

* *Mature miRNA derived from the 5′ arm of the precursor RNA also known as passenger strand*.

### miR-29

miR-29 is the first and most thoroughly investigated miRNA-family in HSCs. miR-29a, miR-29b, and miR-29c are all down-regulated during the *in vitro* activation of isolated rat and mouse HSCs, and in liver biopsies from patients with advanced liver fibrosis. This down-regulation is promoted by transforming growth factor-β (TGF-β) and factors like inflammatory signals including lipopolysaccharide (LPS) and nuclear factor kappa B (NF-κB) (Roderburg et al., 2011). The miR-29 family is of importance for HSC activation, as they can bind to 3′-UTR collagen types I and IV (Kwiecinski et al., 2011). Consequently, miR-29 overexpression in HSCs reduces Collagen I and IV synthesis (Roderburg et al., 2011) and maintenance of the quiescent morphology (Sekiya et al., 2011). In addition to collagen targeting, PDGF-C and IGF-I are identified as targets of miR-29, with PDGF-C having pro-mitogenic and migratory capacities, and IGF-I being an important mitogenic factor when present in an autocrine manner in combination with PDGF-BB (Kwiecinski et al., 2012). In support with these findings, miR-29a/b levels were found to decrease in CCl_4_-treated male mice. Interestingly, female mice do not show this decrease, most likely due to differences in E2, which can induce miR-29a/b levels (Zhang et al., 2012). Not only collagen production, but also other aspects of HSC activation such as inflammatory response and cell proliferation can be regulated by miRNAs such as is the case for miR-146a and miR-16, respectively.

### miR-146

miR-146 is also down-regulated during TGF-β-induced HSC activation (He et al., 2012b), while overexpression of miR-146a in HSCs leads to up-regulation of tissue inhibitor of metalloproteinase 3 (TIMP-3) and down-regulation of IL-6 mRNA (Maubach et al., 2011). In another study, overexpression of miR-146a lead to inhibition of proliferation of activated HSCs. This would be the result of direct binding to the promoter region of the SMAD4 mRNA, which regulates TGF-β1-mediated gene expression, thus leaving the cell insensitive to TGF-β1 stimulation (He et al., 2012b), demonstrating its importance in the inflammatory response, and its link with liver fibrosis. In addition, miR-146a is known to have a role in the inflammatory response during liver reperfusion injury, as it negatively regulates IL-1 receptor-associated kinase 1 (IRAK1) and Toll-like receptor-associated factor 6 (TRAF6), leading to a decrease in pro-inflammatory cytokine production, and by inhibiting the pro-inflammatory NF-κB pathway (Jiang et al., 2014). MiR-126 represents another miRNA that can regulate the NF-κB pathway by suppressing the expression of NF-κB inhibitor alpha (IκBα), thus leading to NF-κB activation (Feng et al., 2015).

### miR-16

miR-16 is another down-regulated miRNA during HSC activation. This miRNA has been shown to inhibit the expression of Cyclin D1, an important regulator of the cell cycle pathway. Expression levels of miR-16 and Cyclin D1 are inversely correlated in activating HSCs. Overexpression of this miRNA in activated HSCs leads to accumulation of the cells in the G0/G1-phase or G0/G1 to S-phase of cell cycle progression (Guo et al., 2009c). In HSCs, miR-16 also acts as an anti-apoptotic regulator in HSCs, by inhibition of B-cell lymphoma 2 (Bcl-2) translation, a known anti-apoptotic gene, leading to the enhanced expression levels of the underlying caspase-pathway consisting of caspases 3, 8, and 9, and thus induction of apoptosis (Guo et al., 2009b).

## Function of stress-responsive pathways and possible contribution of miRNAs during HSC activation

**Figure 2:**
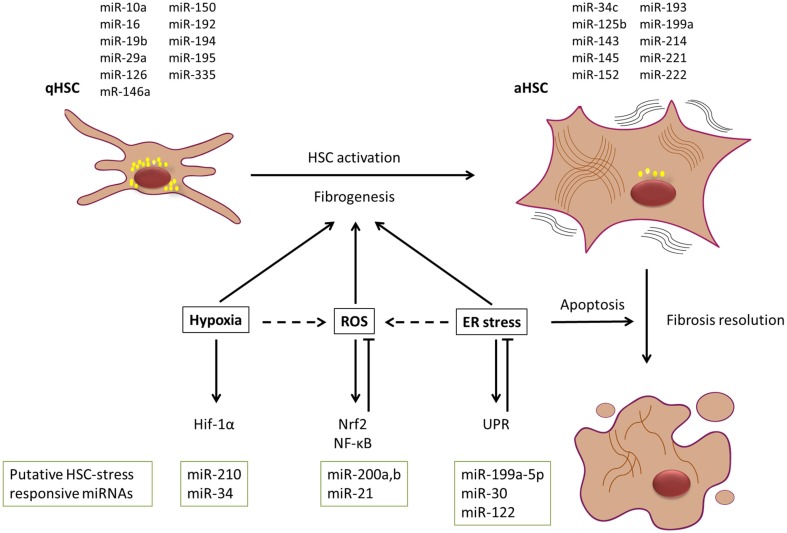
**Dynamic contribution of stress stimuli and miRNAs to liver fibrosis progression and resolution**. HSCs are major contributors to the myofibroblastic cell pool in the fibrotic liver. In the presence of various activation stimuli, HSCs will undergo a myofibroblastic transdifferentiation process toward an activated state, which is characterized by a change in miRNA and mRNA expression pattern. It is widely accepted that the presence of hypoxia, oxidative stress (ROS), and endoplasmic reticulum (ER) stress most likely supports this activation process. However, ER stress could have a potential dual role in the process, as it can also lead to induction of apoptosis in activated HSCs, and thus could contribute to resolution of fibrosis. Simplified representation of some of the signaling cascades and potential miRNAs involved in these stress responses are given. MiRNAs depicted above the HSCs have been reported to be enriched in either qHSC or aHSCs. Putative HSC-stress responsive miRNAs that are discussed in the text are depicted below the signaling cascades.

As mentioned before, HSCs will undergo an activation process in the presence of different (fibrogenic) stimuli like liver injury, paracrine stimulation and autocrine regulation. This activation changes the quiescent fat storing cells into fibrogenic, proliferative and contractile myofibroblasts characterized by their expression of abundant intracellular filaments like α-SMA and vimentin, secretion of ECM including collagen type I and III and fibronectin and their high contractility (Kisseleva and Brenner, 2013). The contribution of stress response pathways in liver fibrosis, cirrhosis and to the HSC activation is generally accepted (Parola and Robino, 2001; Nath and Szabo, 2012; Li et al., 2015), but cannot be interpreted as a simple cause and consequence reaction. As literature mainly describes the contribution of hypoxia (Nath and Szabo, 2012), oxidative stress (Parola and Robino, 2001), and ER stress (Li et al., 2015) pathways during liver fibrosis and cirrhosis progression (Figure 2), we will focus on these three pathways.

Specific stress-related genes can be quickly switched on and off in presence or absence of environmental stress-inducing factors and this can be mediated by miRNAs (Babar et al., 2008; Leung and Sharp, 2010) (Table 2, right panel). So far there are no reports describing the functionality of specific miRNAs in these stress response pathways of activating HSCs during liver fibrosis. However, assumptions about miRNAs forming the link in stress-responsive HSCs (Table 2) and their potential functions in these conditions can be made based on the available data and will be discussed here. We should keep in mind that the presence or lack of overlap in miRNA expression pattern can be due to cell-type and species-specificity and is no proof for actual involvement of the miRNA in stress responsive HSCs, and should be elucidated in future research.

**Table 2:** **Potential miRNAs involved in stress responsive HSC activation**.

	**miRNAs INVOLVED IN HSC ACTIVATION**				**STRESS RESPONSIVE miRNAs IN OTHER CELL TYPES**			
**miRNA**	**Expression during *in vitro* HSC activation**	**Species**	**References**	**Stress**	**Expression during stress**	**Cell type**	**Challenge or treatment**	**References**
miR-214	Up-regulated	Rat, mouse	Maubach et al., 2011; Iizuka et al., 2012	Hypoxia	Up-regulated	Squamous cell carcinoma-cell line	1% oxygen for 1 h or 5% oxygen for 8 h	Hebert et al., 2007
miR-15b	Down-regulated	Rat	Guo et al., 2009b	Hypoxia	Down-regulated	CNE cells: a human naso-pharyngeal carcinoma cell line	Deferoxamine Mesylate	Hua et al., 2006
miR-422b	Down-regulated	Rat	Maubach et al., 2011	Hypoxia	Down-regulated	Squamous cell carcinoma-cell line	1% O_2_ for 1 h or 5% O_2_ for 8 h	Hebert et al., 2007
miR-125b	Up-regulated	Rat	Maubach et al., 2011; Chen et al., 2011	Hypoxia	Up-regulated	Colon and breast cancer cell lines	Culture in 0.2% O_2_	Kulshreshtha et al., 2007
miR-101a	Down-regulated	Rat, mouse	Chen et al., 2011; Tu et al., 2014a	Hypoxia	Down-regulated	Neonatal rat cardiofibroblasts	Culture in 2% O_2_	Zhao et al., 2015
miR-27a	Up-regulated	Rat	Ji et al., 2009	Hypoxia	Up-regulated	Colon-, breast-, human bladder-, and human colon- cancer cell lines	Culture in 3% O_2_, CoCl_2_	Kulshreshtha et al., 2007; Xu et al., 2014
miR-195	Down-regulated	Rat	Maubach et al., 2011	Hypoxia	Down-regulated	Chondrocytes	Culture in 5% O_2_	Bai et al., 2015
miR-210	Up-regulated	Rat	Maubach et al., 2011	Hypoxia	Up-regulated	Pancreatic, breast, head and neck, lung, colon, renal cell lines	2% O_2_ for 24 h	Huang et al., 2009
miR-31	Up-regulated	Rat	Maubach et al., 2011	Hypoxia	Up-regulated	Squamous cell carcinoma-cell line	1% O_2_ for 1 h or 5% O_2_ for 8 h	Hebert et al., 2007
miR-9	Down-regulated	Rat	Ji et al., 2009	Oxidative stress	Down-regulated	ARPE-19: human retinal pigment cells	4-hydroxynonenal and tert-butyl hydroperoxide	Yoon et al., 2014
miR-92a	Down-regulated	Rat	Lakner et al., 2012	Oxidative stress	Down-regulated	TK6: human lymphoblast cell line, endothelial cells (HUVEC)	Irradiation, H_2_O_2_	Chaudhry et al., 2013; Zhang et al., 2014
miR-21	Up-regulated	Rat	Maubach et al., 2011	Oxidative stress	Up-regulated	Neonatal cardiomyocytes	H_2_O_2_	Wei et al., 2014
miR-200a	Up-regulated	HSC-T6 cell line	Sun et al., 2014	Oxidative stress	Up-regulated	Mouse fibroblasts	H_2_O_2_	Mateescu et al., 2011
miR-199a-5p	Up-regulated	Rat	Maubach et al., 2011	ER stress	Up-regulated	Human hepatocyte line	Thapsigargin and deoxycholic acid	Dai et al., 2013
miR-30a	Up-regulated	Rat	Ji et al., 2009	ER stress	Down-regulated	Neonatal rat ventricular cells and rat aorta vascular smooth muscle cells	H_2_O_2_	Chen et al., 2014
miR-122	Down-regulated	Rat	Guo et al., 2009b	ER stress	Down-regulated	Huh7, HepG2 cell lines	Thapsigargin	Yang et al., 2011
miR-30c−2^*^	Down-regulated	Rat	Ji et al., 2009	ER stress	Up-regulated	NIH-3T3 fibroblasts	Tunicamycin and thapsigargin	Byrd et al., 2012
miR-34a	Up-regulated	Rat	Chen et al., 2011	ER stress	Down-regulated	Mouse embryonic fibroblasts	Brefeldin A	Upton et al., 2012
miR-455	Up-regulated	Rat	Ji et al., 2009	ER stress	Down-regulated	Neonatal rat ventricular myocytes	Tunicamycin	Belmont et al., 2012
miR-181a	Up-regulated	Human HSC cell line	Zheng et al., 2015	ER stress	Down-regulated	Various cell lines	Thapsigargin treatment	Su et al., 2013

*MiRNAs which display an overlap in expression profile between activating HSCs and in specific stress responses are displayed in green, those with a contradictory expression profile are displayed in red*.

* *Mature miRNA derived from the 5′ arm of the precursor RNA also known as passenger strand*.

## Hypoxia regulated miRNAs

In the process of liver fibrosis and cirrhosis, hypoxia in the liver cells can be due to disruption of the normal hepatic blood flow, damage of the microvasculature, and excessive deposition of extracellular matrix in the sinusoidal space (Copple et al., 2006). Cellular hypoxia leads to the activation of several Hypoxia Inducible Factors (HIFs), a family of transcriptional factors that work as key regulators for the maintenance of cellular homeostasis when confronted with low oxygen levels (Paternostro et al., 2010). At normal cellular oxygen levels, the oxygen-dependent hypoxia inducible factor HIF-1α (HIF-1α) is hydroxylated by members of the prolyl hydroxylase family (PHD), leading to the rapid degradation of this protein. Decrease of the cellular oxygen levels leads to loss of function of PHD, and subsequent accumulation and translocation of HIF-1α/HIF-2α to the nucleus. In the nucleus, the functional HIF transcription factor complex is formed consisting of HIF-α, HIF-1β and some hypoxic responsive elements (Semenza, 2007). HIF regulates certain processes such as angiogenesis, iron metabolism, glycolysis, and pH control (Jiang et al., 1996; Rosmorduc et al., 1999; Moon et al., 2009). Hypoxic conditions lead to activation of the HSC cell line LX-2 as illustrated by an up-regulation of α-SMA and collagen I protein levels, possibly through activation of the Smad/TGF-β pathway (Shi et al., 2007). HIF is proposed as a main regulator of hypoxia-mediated HSC activation, since it can act as a regulator and stimulator of profibrogenic mediators such as platelet-derived growth factor (PDGF) A and B, plasminogen activator inhibitor-1, and vascular epithelial growth factor (VEGF) (Forsythe et al., 1996; Moon et al., 2009; Wang et al., 2013). The essential role of HIF-1α during hypoxia-induced HSC activation was confirmed *in vitro* by inhibition of HSC-activation due to silencing of HIF-1α (Wang et al., 2013), and the reduced expression of activation genes in HIF-1α-deficient HSCs undergoing hypoxia (Copple et al., 2011). *In vivo* experiments using bile duct ligated (BDL) Hif-1α-deficient and control mice, showed less fibrosis in Hif-1α-deficient mice, as observed by lower levels of α-SMA and type I collagen, thus further indicating its importance during liver fibrosis (Moon et al., 2009).

MiRNAs can act down-stream and up-stream of the HIF pathway. For example, miR-210 expression is directly regulated by HIF-1α as it can bind to the hypoxia responsive element (HRE) located up-stream of the transcription start site of miR-210, leading to its enhanced transcription (Huang et al., 2009). It is suggested, that HIF-2α would mediate miR-210 expression in the absence of HIF-1α, also by interaction with consensus HREs in the miR-210 promoter region (Zhang et al., 2009). MiR-210 effects a broad variety of cellular processes such as fine-tuning cell proliferation by targeting e2f transcription factor 3 (E2f3) (Giannakakis et al., 2008) and MNT, a known MYC antagonist, and a member of the Myc/Max/Mad network (Zhang et al., 2009) while regulating apoptosis by controlling expression of the pro-apoptotic FLICE-associated huge protein (FLASH)/caspase-8-associated protein 2 (Casp8ap2) (Kim et al., 2009). Genes such as Nptx1, Rad52, Acvr1b, Fgrl, Hoxa1, and Hoxa9 associated with pathways like angiogenesis, tumor invasion, regulation of the mitochondrial metabolism, and DNA damage repair were also found to be miR-210 targets (Fasanaro et al., 2009; Huang et al., 2009). The hypoxia-induced up-regulation of miR-210 in various cancer cell lines (Huang et al., 2009) displays an overlap with its enhanced expression during the activation process of HSCs, thus suggesting a potential role of this miRNA in hypoxia-mediated HSC activation.

Another potential link in hypoxia-mediated regulation of HSC activation is presented by miR-31. MiR-31 is up-regulated in both *in vivo* and *in vitro* activated rat HSCs (Maubach et al., 2011). This was confirmed in humans, where miR-31 was not changed in whole liver samples of fibrotic livers, but an increased expression of miR-31 was detected in HSCs during fibrogenesis. Functional studies showed repression of HSC activation by miR-31 inhibition, while miR-31 overexpression revealed its promoting role in cell migration (Hu et al., 2015). Interestingly it has been suggested that the biological function of miR-31 in activating HSCs would be obtained through its effect on Factor-inhibiting HIF-1(FIH) (Mahon et al., 2001; Hu et al., 2015). In head and neck carcinoma, miR-31 negatively regulates the expression of FIH and can thus regulate the expression of FIH in a hypoxia-independent manner (Liu et al., 2010). In cancer models this miRNA is up-regulated under hypoxic conditions (Hebert et al., 2007), suggesting a very complicated and diverse functionality of miR-31 during the reach for cellular homeostasis. Previous research identified a direct link between the increased nuclear levels of HIF-1α protein and an increased activated status of HSCs in a hypoxic environment. HIF-1α has an indirect activating effect on the expression of pro-fibrogenic genes such as TGF-β, IL-6 and CTGF (Copple et al., 2011; Wang et al., 2013). The exact role of miR-31 in this hypoxia-induced HSC activation remains to be elucidated. We speculate on two possible scenarios that are perhaps not exclusive. Due to pro-activating signals from surrounding liver cells, HSCs will up-regulate miR-31 expression, leading to inhibition of FIH function, and thus enhanced HIF-1α expression, thereby favoring HSC activation in normoxic conditions. Hypoxic regions appear in the liver due to injury, what could favor the (further) induction of miR-31 expression, boosting the already enhanced HIF-1α expression, further leading to progression or maintenance of HSC activation.

## Oxidative stress regulated miRNAs

Cells in aerobic organisms have a continuous balance between the production of pro-oxidants, such as reactive oxygen species (ROS), and anti-oxidants. When a cell is subjected to oxidative stress, this normal balance fades by excessive production of pro-oxidants. Various types of ROS are known, such as the singlet molecular oxygen, hydrogen peroxide and the hydrogen radical, which all have a specific half-life and mechanism of action (Sies, 1991).

There are several possible sources of ROS in the cell. Mitochondria, the main site of oxygen consumption in aerobic cells, are the main producers of ROS derived mainly through the leakage of electrons and formation of superoxide (Guarente, 2008). Cytochrome P450 (CYP) acts in the detoxification of metabolic as well as xenobiotic compounds by means of oxidation (Aubert et al., 2011) making it also an important source of ROS. Specifically the form CYP2E1, which is highly expressed in hepatocytes, has been demonstrated to be a key source of ROS in the liver (Poli, 2000). Another major source of ROS in several cell types and HSCs is nicotinamide adenine dinucleotide phosphate-oxidase (NADPH oxidase) (De Minicis and Brenner, 2007; Sergey, 2011).

Oxidative stress and the subsequent decreased levels of anti-oxidants during liver fibrosis has been shown for a broad variety of etiologies (Poli, 2000). ROS are produced by various cell types, but it is thought that the major contributors of ROS production in this pathology are apoptotic hepatocytes. HSCs express a non-phagocytic form of NADPH oxidase, which presents a basal level of activity, producing constitutively low levels of ROS and increasing production upon different stimuli (Bataller et al., 2003). NADPH oxidase of HSCs is activated upon phagocytosis of these apoptotic bodies of hepatocytes (Shan-Shan et al., 2006). Furthermore, NADPH oxidase-generated ROS in HSCs is also induced by advanced glycation end-products (AGEs) which are products of a non-enzymatic reaction of sugars with molecules such as proteins, lipids and nucleic acids that accumulate in diseases related to the metabolic syndrome (Yan et al., 2010). Liver fibrosis is correlated with accumulation of systemic AGEs and ROS in HSCs has been show to participate during the development of liver diseases (Šebeková et al., 2002; Hyogo et al., 2007; Guimarães et al., 2010).

Activated Kupffer cells and neutrophils are also described as important producers of ROS during early stages of liver fibrosis (Kisseleva and Brenner, 2007). The most important result of oxidative stress is lipid peroxidation. As example, liver fibrosis caused by excessive alcohol intake leads to injury of the different liver cell types and consecutive excessive oxidation of polyunsaturated membrane lipids due to enhanced generation of ROS due to the elevated levels of cytochrome CYP2E1 (Nieto et al., 1999). The products of such lipid peroxidation could further catalyze the progression of fibrosis by activation of the production of collagen α2 (I) in HSCs in a paracrine manner (Bedossa et al., 1994). Furthermore, exposure of HSCs to ROS can promote their proliferation and invasiveness. It is thought that it would obtain these effects by an induction of MMP-2 expression, and the enhancement of MT1-MMP and TIMP-2 protein levels, in an ERK1/2 and PI3K dependent manner (Galli et al., 2005).

Several miRNAs have already been linked to the regulation of the oxidative stress pathway, including members of the miR-200 family. From this miRNA-family, especially miR-200c has been shown to display an increased expression after cellular exposure to H_2_O_2_. This miRNA would lead to down-regulation of zinc finger E-box binding homeobox 1 (Zfhx1a, aka Zeb1, or TCF8), a transcriptional repressor, both on mRNA and protein level, leading to cellular senescence and inhibition of cell proliferation. Interestingly, an inhibitory loop was found between miR-200c and Zeb1, as the promoter region of miR-200c contains two conserved Zeb1 binding sites (Magenta et al., 2011). MiR-200c can also regulate apoptosis, as it inhibits the translation of FAS associated phosphatase (FAP-1) mRNA. Decreased expression of FAP-1 leads to a greater sensitivity to CD95-mediated apoptosis (Schickel et al., 2010). Some of the other identified targets of miR-200c include Moesin (MSN), Fibronectin 1 (FN1), and Rho GTPase activating protein 19 (ARHGAP19), important regulators of the migratory and invasive capacity of cancer cells (Howe et al., 2011). Another miRNA associated with oxidative stress is miR-21. Cells exposed to ROS would up-regulate miR-21, which can directly interact with the 3′UTR of the programmed cell death 4 (PDCD4) gene, a known tumor suppressor and apoptosis-regulator, thereby preventing cell death. Oxidative stress mediated up-regulation of miR-21 can be induced by NF-κB activation through five NF-κB binding sites in the 5' miR-21 promoter region (Tu et al., 2014b; Wei et al., 2014). Up-regulation of miR-21 would be a down-stream effect of NADPH oxidase activity (Dattaroy et al., 2015), as this induces NF-κB translocation to the nucleus (Yao et al., 2007) and its subsequent binding to the miR-21 promotor (Sheedy et al., 2010). This enhanced expression of miR-21 also leads to a suppression of SMAD7 expression and therefore favors assembly of SMAD2/3-SMAD4 heterodimers, a crucial event in the pro-fibrogenic TGF-β signaling pathway (Dattaroy et al., 2015).

A potential link in oxidative stress-induced HSC activation could be represented by miR-200a, which is down-regulated during the process of liver fibrosis in rat, and in TGF-β1-mediated activation of a rat HSC cell line (Sun et al., 2014). MiR-200a also regulates proliferation of these activating HSCs, shown by an accumulation of cells in the G0/G1 phase upon miR-200a overexpression. Targets of miR-200a include pro-fibrogenic factors TGF-β2 and β-catenin (Sun et al., 2014). Another important miRNA-200a target gene is Kelch-like ECH-associated protein 1 (Keap1), which negatively regulates the stability of nuclear factor-erythroid-2-related factor 2 (Nrf2), a known regulator of the expression of antioxidants involved in the protection against oxidative damage (Yang et al., 2014). While no information is available for miR-200c, in rat, miR-200a seems to be down-regulated upon HSC activation while during liver fibrosis progression in human and mouse, miR-200a and miR-200b undergo a significant up-regulation (Murakami et al., 2011). This is in line with the up-regulation in expression of the miR-200 family after induction of oxidative stress in mouse fibroblasts where miR-200a can target p38α mitogen-activated protein kinase (MAPK) (Mateescu et al., 2011), which is downstream of the oxidative stress stimulus, and leads to an inhibition of cell division (Kurata, 2000). Despite opposing expression patterns observed in different species, the involvement of miR-200a in both HSC activation and oxidative stress response is clear. It is therefore tempting to speculate that miR-200a could participate in the anti-oxidant response of HSCs during liver injury.

## Endoplasmic reticulum stress regulated miRNAs

The generation of mediators that lead to a perturbation of the ER homeostasis can be evoked by various stimuli associated with the initiation or progression of the liver fibrosis process, such as repeated cycles of ischemia and reperfusion due to distorted hepatic flow, genetic mutations of proteins involved in ER constitution and function, excessive exposure to certain drugs (paracetamol, ethanol), obesity-linked enhanced presence of lipids, and viral infections (HCV, HBV). These stimuli can lead to oxidative stress, formation of protein aggregates, altered membrane lipid-composition, and hyperhomocysteinemia with resulting N-homocysteinylation, all leading to the dysfunction of the ER, and accumulation of unfolded and misfolded proteins (Malhi and Kaufman, 2011). Cells will try to counteract this accumulation of misfolded proteins by diverse mechanisms such as the unfolded protein response (UPR). The activation of the UPR pathway, due to ER-resident stress sensors such as ATF-6, IRE1, and PERK (Asselah et al., 2010), will lead to an enhanced and more stringent folding and degradation of proteins in the ER, and an overall diminishment of protein synthesis. When the UPR fails to diminish the ER stress, the cells go into apoptosis. Persistent ER stress has several consequences including the excessive energy depletion due to the enhanced utilization of energy for translocation of misfolded proteins; ASK1/JNK mediated signaling leading to activation of caspases, and the activation of the pro-apoptotic pathway of CHOP/GADD153 transcription factor, which all direct the cell toward apoptosis (Xu et al., 2005). It will also lead to the release of the stored calcium in the ER, which affects mitochondria; moreover it will lead to the induction of oxidative stress, activation of the pro-inflammatory NF-κB pathway and apoptosis of the cell. ER stress will also lead to translocation and activation of SREBP, causing an enhanced synthesis of lipids such as fatty acids and cholesterol, and an enhanced cellular uptake of lipoproteins (Ji and Kaplowitz, 2006; Ji, 2008).

Cultured HSCs, which are known to be relatively apoptosis-insensitive, have been shown to undergo apoptosis in response to persistent ER stress due to an increase of the amount of intracellular calcium, and activation of JNK/p38 MAPK and Calpain/Caspase pathways (Huang et al., 2014b). Activation of the latter pathway can be explained by the decrease of Calpastatin expression, which works as an inhibitor of the pro-apoptotic Calpain. During the activation of HSCs, Calpastatin levels become elevated, leading to the desensitization of the HSCs toward apoptotic stimuli. ER-stress mediated decrease of Calpastatin expression can thus lead to higher Calpain levels, and consequent sensitization toward apoptotic stimuli (De Minicis et al., 2012). The fibrosis counteracting effect of ER stress was further supported by the decrease in α-SMA and Col1a1-expression in ER-stress responsive activating HSCs (Huang et al., 2014b). However, it is found that when HSCs are exposed to oxidative stress-induced ER stress, the UPR will lead to the up-regulation of different pathways leading to enhanced autophagy and consequent HSC activation *in vitro* (Hernandez-Gea et al., 2013). All described ER stress could thus be considered as a complex mechanism of fibrosis regulation, with a possible stimulatory role in HSC activation and a possible role in fibrosis resolution due to its pro-apoptotic effects in activated HSCs.

The role of miRNAs during ER-stress remains largely unknown. One of the miRNAs that has been studied in this process is miR-199a-5p, which displays an up-regulation in hepatocytes undergoing ER stress. This miRNA would have several ER-stress related targets including the chaperone protein GRP78 (which is also known as Bip and HSPA5), activating transcription factor 6 (ATF6), and inositol-requiring enzyme 1α (IRE1α), with the latter two being UPR transducers. As IRE1α activated ER stress can induce cell death, activation of miR-199a-5p, and thus subsequent down-regulation of IRE1α, would work as a rescue mechanism to prevent the induction of apoptosis. *In silico* target prediction identified DNA-damage regulated autophagy modulator 1 (DRAM1) and cyclin-dependent kinase inhibitor 1B (p27), both pro-apoptotic genes, as additional potential targets of miR-199a-5p, thus further underlining its pro-survival role (Dai et al., 2013). miR-199a-5p could also have some effect on cell proliferation, as it has been shown to target frizzles type 7 receptor (FZD7), and thus regulates the expression of its downstream genes including β-catenin, Jun, Cyclin D1, and Myc (Song et al., 2014). A second class of miRNAs linked with ER stress includes members of the miR-30 family, which are being down-regulated due to this specific stress responsive pathway. This miRNA family contains six members (from a to e), which contain all an identical seed sequence motif, but are located at different sites of the genome. GRP78 is targeted by miR-30a, which further underlines the importance of this miRNA in this stress response. Knockdown of miR-30 in cardiac cells identified ATF6, CHOP, and caspase-12 as indirect targets of this miRNA, thus revealing its role in regulation of cell death (Chen et al., 2014).

MiR-122 could perhaps represent a regulator of ER-stress-modulated HSC activation. MiR-122 is described as liver-specific and the most abundant miRNA in the liver (Lagos-Quintana et al., 2002). It has been shown that miR-122 is down-regulated in total liver samples during the progression of liver disease in mouse, rat (Li et al., 2013) and human (Padgett et al., 2009), and this down-regulation was furthermore observed in activating HSCs (Li et al., 2013). Overexpression of this miRNA in LX-2 cells leads to a decrease in cell proliferation and maturation of Col1a1, most likely through regulation of P4HA1 by miR-122. The expression of P4HA1 is up-regulated during fibrosis progression, and encodes a component of prolyl 4-hydroxylase, which is necessary for collagen maturation (Li et al., 2013). Overexpression of miR-122 in LX2 further identified FN1, which is involved in the assembly of collagen fibrils, and serum response factor (SRF) as direct targets, and confirmed its inhibitory effect on TGF-β-induced HSC activation (Zeng et al., 2015). Further target identification studies in hepatocytes identified mitogen-activated protein kinase kinase kinase 3 (MAP3K3), which plays a role in cell survival and proliferation, the intermediate filament vimentin, and HIF-1α (Csak et al., 2015). MiR-122 inhibition in hepatoma cells suggests a role in the UPR. Moreover its inhibition leads to an up-regulation of the 26S proteasome non-ATPase regulatory subunit 10 (PMSD10), which can enhance the protein folding-capacity and thus promoting recovery, by up-regulation of GRP78. MiR-122 would have this effect on PMSD10 in an indirect manner through targeting of cyclin dependent kinase 4 (CDk4) which interacts with PMSD10. Other miR-122 targets include the ER stress chaperones calreticulin (CALR), ER protein 29 (ERP29) and SET nuclear oncogene (SET), which help in the correct folding of malfunctional proteins (Yang et al., 2011). Taken together, even though miR-122 is not abundantly expressed in HSCs, it is tempting to speculate that down-regulation of miR-122 is involved in the UPR in HSCs.

## Discussion

MiRNAs have been proposed as key regulators of gene expression and dysregulated patterns of miRNA expression were observed in various diseases (Tufekci et al., 2014), including the progression of liver fibrosis and cirrhosis (Wang et al., 2012; Xin et al., 2014). Studying miRNAs is very popular and raised a lot of expectations in their use as biomarkers for diseases and therapeutic interventions using miRNA mimics and antagomirs. Unfortunately, so far this has not turned out to be easy, partly because of their cell type-specific and species-specific activity and wide range of targets.

Diagnosis of liver fibrosis could be facilitated by identification of blood-circulating biomarkers representative for HSC activation, as the current golden standard for diagnosis remains the invasive and harmful liver biopsy (Piccinino et al., 1986; Friedman, 2003). Circulating miRNAs, both protein-bound and packaged into extracellular vesicles (Turchinovich et al., 2011), have been proposed as such a potential biomarker, and various research groups already tried to identify circulating miRNAs that could be linked with progression and regression of liver disease (Roderburg and Luedde, 2014). To date, this has not yet led to a diagnostic protocol that is used in clinic. It is tempting to speculate that perhaps stress-responsive miRNAs of activating HSCs secreted in the blood could also be used as a liquid biopsy to document the stress present in the liver.

We discussed several miRNAs with a potential role in stress-mediated regulation of HSC activation. Experimental validation of these suggested links between stress-related miRNAs and HSCs should address a number of issues. First, are specific miRNAs dysregulated in HSCs in response to specific stress signals and does this lead to an imbalance of the cellular homeostasis and consequent HSC apoptosis or activation? *In vivo*, paracrine stimulation of quiescent HSCs by stress-undergoing surrounding cells is likely to create a warning for the quiescent cell, leading to its activation and reducing its responsiveness to more stress-signals. Secondly, responding to stress is necessary to counteract short term challenges to restore cell homeostasis. Thus the question is, whether there are miRNAs that specifically respond to prolonged stresses present in the fibrotic liver, and if so, could a targeted mimic/antagomir approach inhibit HSC activation or promote HSC apoptosis or inactivation?

In conclusion, HSC activation *in vivo* can be seen as a very complicated and multifactorial process in which hypoxia (Cannito et al., 2014), oxidative stress (Poli, 2000), and ER stress (Malhi and Kaufman, 2011) are surely involved. This suggests a potential role for stress-related miRNAs during HSC activation and disease development and opens perspectives for new therapeutic approaches.

### Conflict of interest statement

The authors declare that the research was conducted in the absence of any commercial or financial relationships that could be construed as a potential conflict of interest.
